# Strengthening retina eye care services in Nepal: retina eye care of Nepal project

**DOI:** 10.1186/s12913-020-05850-x

**Published:** 2020-10-27

**Authors:** Arjun Shrestha, Chunu Shrestha, Pratap Karki, Hara Maya Gurung, Takeshi Naito

**Affiliations:** 1 BP Eye Foundation, Hospital for Children, Eye, ENT & Rehabilitaion Services, Bhaktapur, Nepal; 2 Nepal Eye Hospital, Kathmandu, Nepal; 3 BP Koirala Lions Club of Ophthalmic Studies, Kathmandu, Nepal; 4 Himalaya Eye Hospital, Kaski, Nepal; 5 Department of Ophthalmology, Institute of Biomedical Sciences, Tokushima University Graduate School, Tokushima, Japan

**Keywords:** Retina eye care, Nepal, Strengthening

## Abstract

**Background:**

Retinal diseases are very difficult to treat. So, early diagnoses and preventions are very important. But, few eye doctors can treat patients with retinal diseases in Nepal. Retina Eye Care of Nepal (RECON) project was designed to strengthen retina eye care services in Nepal.

**Methods:**

RECON was implemented from May 2016 to February 2019 in Nepal. Four Master Eye Doctors (MED) received Training of Trainers (TOT) from Tokushima University, Japan. MEDs developed training materials for different cadres of ophthalmic human resources, enhanced retina eye care facilities, and conducted retina-screening camp in Nepal.

**Results:**

Twenty ophthalmologists, 16 optometrists, 48 ophthalmic assistants and 17 ophthalmic nurses, 76 physicians and 28 health workers were trained in retina care. Eight outreach retina camps were conducted.

**Conclusions:**

The project was a novel approach to strengthen retina services in Nepal. The aim of the project was accomplished with the ultimate benefits to the needy retina patients who otherwise were going to miss the retina services.

## Background

Recently causes of blindness are changing in Nepal. The number of people blind due to retinal diseases is increasing. Age-related macular degeneration (AMD), diabetic retinopathy (DR), hypertensive retinopathy, and retinal vein occlusion are the major retinal problems in Nepal. As the prevalence of vitreoretinal disorders is increasing with age, it indicates that retinal disorders will be a major public health issue with longevity in future [1].

A rapid assessment of blindness conducted in 2010 had reported posterior segment problems as the second common cause of blindness, after cataract in Nepal [2]. Retinal diseases are very difficult to treat. Results from low-income countries show that many patients present only when they lose vision in both eyes. Delay in presentation was acknowledged as a significant problem and is often due to inadequate primary eye care and to misdiagnosis. Thus, it was highlighted that all ophthalmologists should be trained to recognize and manage retina problems. As blindness from DR is preventable, if caught and treated early, DR provides an excellent opportunity for secondary prevention strategies, such as screenings [3]. So, early diagnoses and preventions are very important.

The projected population of Province 3 and 4 in Nepal were 60, 26,626, and 24, 72,494 respectively in the year 2016 [4, 5]. There are altogether 6 tertiary retina care centers in Province 3 and 1 tertiary retina center in province 4 of Nepal to cover that much of the population. But, few eye doctors can treat patients with retinal diseases in Nepal. It is also necessary to train ophthalmic assistants, optometrists, ophthalmic nurses, and other health workers who can assist the treatment of retinal diseases. So, strengthening retina eye care services is very important in Nepal.

Retina Eye Care of Nepal (RECON) project was a joint program of BP Eye Foundation, Kathmandu, Nepal, and Tokushima University, Japan to strengthen 3 retina centers in Province 1 and 1 retina center in province 4. The project duration was from May 2016 to February 2019. The purpose of RECON was to strengthen retina eye care services in Nepal by training ophthalmic human resources, enhancing retina eye care facilities, and conducting retina-screening camp.

## Methods

**Fig. 1:**
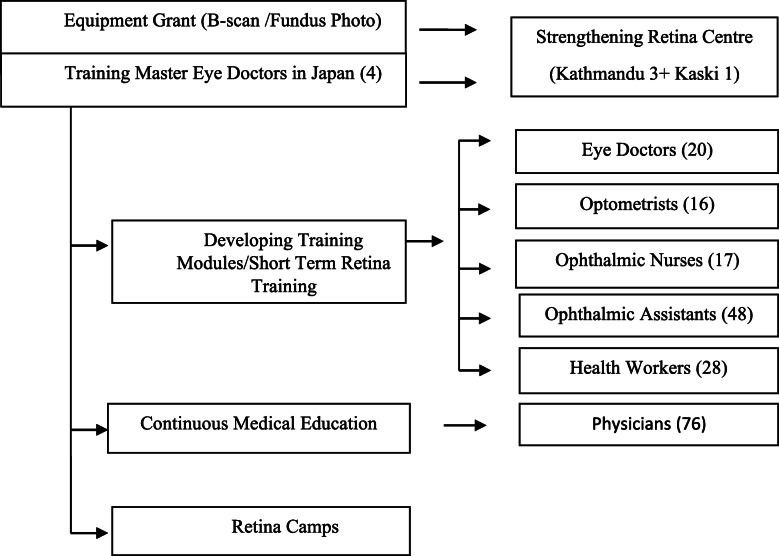
Algorithm of RECON activities. RECON = Retina Eye Care of Nepal

RECON established a unique approach to accomplish its objectives (Fig. 1).

### Master eye doctor (MED) training

Four eye institutes, Hospital for Children, Eye, ENT & Rehabilitation Services (CHEERS) located at Bhaktapur district, Himalaya Eye Hospital (HEH) located at Kaski district, Nepal Eye Hospital (NEH) and BP Koirala Lions Club of Ophthalmic studies (BPKLCOS), located at Kathmandu district were selected as the partner retina centers. CHEERS and HEH had one retina specialist each and NEH and BPKLCOS had two retina specialists each at the time of project execution. Four Retina specialists, one from each partner retina center of Nepal received one-month Training of Trainers (TOT) in retina care from Tokushima University of Japan in the first year of the project and got a certificate of Master Eye Doctor (MED).MED also received advanced retina training for 2 weeks at Kindai University and the Tokushima University of Japan at the beginning of the 3rd year of the project.

### Strengthening of retina Centres

All partner retina centers already had vitrectomy machines and laser facilities. Vitreoretinal procedures were already in functional states at all retina centers except at CHEERS. Moreover, BPKCLOS and NEH located at the capital city already had residency programs. HEH is located outside of the capital city while CHEERS is a new hospital at Bhaktapur, within close premises of the capital city. The project supported fundus photography machines, ophthalmic ultrasonic imaging, wide-angle viewing system, and cryo machine to these partner eye institutes. MEDs started strengthening of retina clinics with knowledge and skills gained during TOT in retina care from Tokushima University.

### Advocacy and retina force networking

MEDs conducted first continuing medical education (CME) on retina eye care with facilitators and visiting retina experts from Tokushima University to the targeted ophthalmologists with a focus on retina force networking. Second CME was on developing vitrectomy in Nepal.

### Training human resources

These MEDs prepared training materials on retina management for different cadre of ophthalmic human resources including general ophthalmologists, optometrists, ophthalmic assistants, ophthalmic nurses, and female community health volunteers. These MEDs already provided two weeks retina training for ophthalmologists, one-week training for optometrists, ophthalmic assistants, and ophthalmic nurses from different eye institutes of Nepal. The training included early detection and prompt referral of retinal diseases from the community to the retina training centers.

### Retina camps

#### Outreach retina camps were conducted outside of base hospitals to target the community who otherwise would not visit the base hospital due to various reasons

Portable fundus camera, binocular and monocular indirect ophthalmoscope with 20 dioptre convex lenses were used to screen the retinal diseases at eye camps. The patients with retinal diseases found at eye camps were sent to the respective retina centers for further treatment. Retina camps were conducted in partnership with local health clubs, municipalities, or nongovernment organizations. Each camp shared the task among different cadres of health care workers including MED, optometrist, ophthalmic nurse and ophthalmic assistant. MED performed fundoscopy and advised for further treatment, optometrist performed fundus photography, ophthalmic nurses helped in counseling and ophthalmic assistant helped in taking vision, pupil dilation, and history taking.

## Results

Table 1 shows the number of vitreo retina procedures by all 4 retina centers before and during the project period. There were no vitreoretinal procedures performed at CHEERS before the RECON project. The vitreo retina procedures were performed more in already established partner eye institutes; NEH and BPKLCOS. NEH and BPKCLOS are located in the capital city, already had two retina specialists and the experiences of residency programme. All retina centers are doing good performance every year after enrolling in the RECON project. These retinal procedures were invariably performed by the retina specialists of the respective retina centers. Advocacy campaigns and retina camps were held as shown in Table 2.

**Table 1:** Vitreo retinal procedures

	Before RECON		During RECON			
Vitreo Retinal Surgeries	Year 1	Year 2	Year 1	Year 2	Year 3	
	0	0	18	147	211	CHEERS
	51	102	160	167	260	BPKLCOS
	44	96	210	314	349	NEH
	14	27	38	45	48	HEH
Intravitreal injections	0	0	64	301	402	CHEERS
	0	93	374	370	583	BPKLCOS
	72	188	367	773	1544	NEH
	305	314	411	686	1088	HEH
Retinal Lasers	0	0	17	113	123	CHEERS
	65	79	178	290	336	BPKLCOS
	55	73	168	250	448	NEH
	25	35	78	99	105	HEH

*BPKCLOS* BP Koirala Lions Club of Ophthalmic studies, *CHEERS* Hospital for Children, Eye, ENT & Rehabilitation Services, *HEH* Himalaya Eye Hospital, *NEH* Nepal Eye Hospital, *RECON* Retina Eye Care of Nepal

**Table 2:** Advocacy and Retina camps

**No of CMEs**	**2**
**No of Retina Camps**	**8**
**No of Retina patients screened at the camp**	**627**
**No of IEC materials distributed**	**2000**

*No* Number, *CME* continuous medical education

*IEC* information, education & communication

## Discussion

Recently, retinal disease is also regarded as a public health concern in the developing world. There have been improved and cost-effective treatment options available for retinal problems even in a developing world. However, a significant challenge still is the shortage of skilled human resources. There is an observed and felt need for more ophthalmic health force with subspecialty training in retinal disease who can train future generations of eye workers in this mid and low-income countries [6].

A significant barrier was the practice of setting up a screening system without adequate treatment facilities being in place [3]. In our case, all retina centers already had vitrectomy machines, laser facility, and optical coherence tomography. RECON executed skill enhancement, trained, and retrained MEDs of Nepal in capacity building. CME helped in advocacy campaign and retina forces networking. All the trainees, eye doctors, optometrists, ophthalmic assistants, ophthalmic nurses, and physicians were providing education to retina patients about the importance of retina check-up and referring them in need to our retina care centers.

The general practitioners and physicians are often the first medical personals to see the patients with diabetes. There are varying levels of awareness about diabetic retinopathy among physicians. This is very crucial as they are the ones referring patients with diabetes to ophthalmologists. It is not surprising to see an unsatisfactory level of knowledge and awareness of physicians about the importance of diabetic retinopathy evaluation. Physician’s low level of awareness about diabetic retinopathy is found even in resource-rich settings, and further training is recommended [7–9].

Most Eye camp is widely practiced in all over Nepal. To add the retinal disease screening at eye camps is very useful for the prevention of retinal diseases. Retina camps were the means of eye health education, eye examinations, fundus photography to patients in our project.

The conclusion from Bhakapur retina study in Nepal highlighted the real world scenario about retinal diseases in Nepal. The high prevalence of retinal diseases, low awareness on major blinding retinal diseases in the population and high risk groups warrants the prompt attention for awareness campaigns, retinal diseases screening, using allied ophthalmic personnel and allied medical personnel using fundus cameras, proper referral network to tertiary eye hospitals, cross referral with the physician for diseases like diabetes, and hypertension and facilities for treatment of these diseases are required for the prevention of avoidable blindness from these major retinal disorder [10].

In our retina camps, apart from direct beneficiaries who got free consultation in retina camps, there were many indirect beneficiaries in the community. Awareness of diabetic and hypertensive retinopathy and other retinal diseases were the main aims of retina camp. This is a good step of advocacy for early detection and prompt treatment to prevent retina related visual blindness in Nepal.

The comprehensive campaigns are necessary to promote increased awareness in a community by involving people from various walks of life in collaboration with community eye centres and eye hospitals. Improving awareness will help in early detection of diseases and reduction in visual impairment and blindness [11].

## Conclusions

RECON was a novel approach to strengthen retina services in Nepal. The aim of the project was the ultimate benefits to the needy retina patients who otherwise were going to miss the retina services. There was a strengthening of retina eye care of all these retina centers. The project focused on the training retina workforces, enhancing retina care by easy access and improved services and awareness to even grass root level in the community by conducting retina-screening camp. We recommend further similar type of strategy in different provinces of Nepal to combat retinal-related blindness in the future.

## Abbreviations

AMD: Age related macular degeneration
BPKLCOS: BP Koirala Lions Club of Ophthalmic Studies
CHEERS: Hospital for Children, Eye, ENT & Rehabilitation Services
HEH: Himalayan Eye Hospital
NEH: Nepal Eye Hospital
CME: Continuous medical education
DR: Diabetic retinopathy
IEC: Information education and communication
MED: Master Eye Doctor
RECON: Retina Eye Care of Nepal
TOT: Training of Trainers
